# Perspectives on Peritoneal Dialysis and Kidney Transplant in Adolescents and Young Adults: A Qualitative Study

**DOI:** 10.1016/j.xkme.2026.101316

**Published:** 2026-03-11

**Authors:** Alexandra Bicki, Kimberly Koester, Gabriela Accetta Rojas, Elaine Ku

**Affiliations:** 1 Division of Pediatric Nephrology, Department of Pediatrics, UCSF, San Francisco, CA; 2 Division of Prevention Science, Department of Medicine, UCSF, San Francisco, CA; 3 Nemours Children's Hospital, Wilmington, DE; 4 Division of Nephrology, Department of Medicine, UCSF, San Francisco, CA; 5 Department of Epidemiology and Biostatistics, UCSF, San Francisco, CA

**Keywords:** Adolescent health, adolescent and young adult (AYA), chronic kidney disease, decision making, directed donation, end-stage renal disease (ESRD), end-stage kidney disease (ESKD), health care transition, home dialysis, kidney failure, living kidney donation, live donor, peritoneal dialysis, qualitative research

## Abstract

**Rationale & Objective:**

Peritoneal dialysis and kidney transplantation confer better quality of life than hemodialysis, but uptake is low in adolescents and young adults. Our objective was to explore perspectives on peritoneal dialysis and living donor kidney transplantation among adolescents and young adults with chronic kidney disease.

**Study Design:**

A qualitative study using semistructured interviews.

**Setting & Participants:**

Thirteen adolescents and young adults with nondialysis-dependent chronic kidney disease and 10 of their parents were recruited from a single academic medical center in the San Francisco Bay Area, California, United States.

**Analytical Approach:**

Interview transcripts were analyzed qualitatively to identify prevalent themes.

**Results:**

Perspectives on peritoneal dialysis included the following: (1) lack of awareness of peritoneal dialysis as a treatment option; (2) negative impressions of dialysis based on anecdotes from people receiving hemodialysis; and (3) infectious concerns regarding home dialysis. Perspectives on living donor kidney transplantation included the following: (1) resistance to an illness-centered identity; (2) misperception and confusion regarding the waitlisting process; (3) “heavy guilt”: the burden of responsibility for adverse outcomes after directed donation; and (4) ensuring living donor availability for the future. An overlapping theme of reliance on mothers for chronic kidney disease caregiving and potential donation was also identified.

**Limitations:**

This single-center study included patients referred to 1 kidney transplant center, and findings may be context specific.

**Conclusions:**

Adolescents and young adults with chronic kidney disease and their parents had low awareness of peritoneal dialysis and low levels of engagement in the search for donors. Some barriers were unique to this population and differed from those previously identified among older adults with kidney failure. Providers should be aware of these differences when counseling this younger population on kidney replacement therapy options.

## Plain-Language Summary

Peritoneal dialysis and kidney transplants may offer a better day-to-day life for young people with chronic kidney disease compared with in-center hemodialysis. However, many adolescents and young adults do not receive peritoneal dialysis or a living donor kidney transplant. To understand why, we interviewed adolescents and young adults with chronic kidney disease and their parents. We found that many of them had never heard of peritoneal dialysis, were confused about the kidney donation process, or felt guilty accepting a kidney from someone they knew. These findings are important because younger patients have different concerns than older adults with kidney disease regarding various kidney failure treatments, and education on treatment modalities should address the specific concerns of younger patients.

Kidney replacement therapy (KRT) is life-sustaining for individuals with kidney failure, and living donor transplant is considered the preferred form of KRT because of better survival^1^ and quality of life for patients compared with dialysis.^2^ If dialysis must be pursued, peritoneal dialysis (PD) rather than in-center hemodialysis (HD) is the preferred treatment modality recommended for children and young adults given the ability to receive therapy at home, promote school attendance or employment, and experience a superior quality of life.^3^^,^^4^ Rates of both PD initiation and receipt of a living donor kidney transplant decrease with age. In-center HD remains the most common initial KRT modality in adolescents and young adults, with 57% of adolescents and over 80% of adults initiating in-center HD at the onset of kidney failure.^1^

The reasons for the low uptake of PD and living donor kidney transplant in the adolescent and young adult population are unclear, especially given these patients’ high likelihood of having parents or guardians who could be available for directed donation or as support for home dialysis. Prior qualitative studies around KRT decision making in adolescents and young adults have largely been conducted outside the United States and include patients already treated with dialysis or transplant.^5^, ^6^, ^7^, ^8^ Prior themes that informed decision making in this younger population included identity disruption, decisional uncertainty, and efforts to preserve their sense of normalcy. However, less is known about how patients with nondialysis-requiring chronic kidney disease (CKD) and their families understand and anticipate KRT options before the onset of kidney failure. Thus, the objective of this study was to ascertain perspectives on KRT modalities among adolescents and young adults with nondialysis-requiring CKD and their parents in the United States.

## Methods

Between July 2023 and November 2024, patient–parent dyads were invited to participate in semistructured interviews to elucidate their perspectives on living donor kidney transplant and home dialysis (primarily focusing on PD, as home HD is rare in pediatric practice). The University of California, San Francisco (UCSF) Institutional Review Board approved this study, and informed consent (or assent with parental consent for patients <18 years) was obtained from all participants. This study followed the Consolidated Criteria for Reporting Qualitative Research reporting guidelines (see Item S1 for details).^9^

### Participant Eligibility and Recruitment

Patients were eligible if they met the following criteria: (1) between 14 and 29 years of age at the time of interview; (2) had childhood-onset CKD; (3) had current CKD stage 3b-5 (defined as most recent glomerular filtration rate <45 mL/min/1.73 m^2^ as calculated using the Schwartz equation or as documented in their nephrologist’s most recent clinical note); (4) had been evaluated for kidney transplantation at UCSF, which facilitated recruitment and confirmed the patient’s need for eventual KRT; and (5) spoke English or Spanish. The lower age limit includes adolescents as defined by the American Academy of Pediatrics, whereas the upper age limit includes young adults with kidney failure defined by others in prior quantitative^10^^,^^11^ and qualitative^6^, ^7^, ^8^ analyses. Patients were excluded if they had received any prior form of KRT.

Eligible patients were identified through electronic health record screening and provider referral and then invited to participate via phone, text, or patient portal messaging (first sent to the parent, if the patient was a minor). Parents (identified by the patient) were eligible for participation in interviews either independently or jointly with the patient. Although nonparent caregivers were eligible to participate, all patients invited a parent.

### Data Collection

A semistructured interview guide informed by prior studies^12^, ^13^, ^14^, ^15^, ^16^ was developed by AB and revised by EK and KK. The guide explored (1) comprehension and expectations surrounding treatment options for kidney failure; (2) attitudes toward the dyad’s preferred dialysis modality, if chosen; and (3) attitudes toward transplant and living donation (Item S2).

Participants completed a one-time interview with AB, either virtually or in person based on participants’ preference. Interviewing as a dyad was not compulsory; patients and parents who elected to complete part of the interview as a dyad were also given time to discuss their experiences with AB independently. Participants were offered a $60 gift card in appreciation of their time. Interviews were recorded and audio/video files were uploaded to MAXQDA (Berlin, Germany) and transcribed then deidentified by AB.

### Analytic Approach

This study was informed by grounded theory principles to develop a conceptual understanding from participants’ perspectives,^17^ with analysis operationalized using a sort and shift approach^18^ emphasizing iterative, data-driven theme development (see Item S1). AB, GAR, and EK independently coded transcripts; KK reviewed a subset of transcripts to provide methodological guidance and ensure rigor. Interviews continued until thematic saturation was reached, defined as 2 successive dyad interviews yielding no substantively new conceptual insights.

## Results

**Figure 1:**
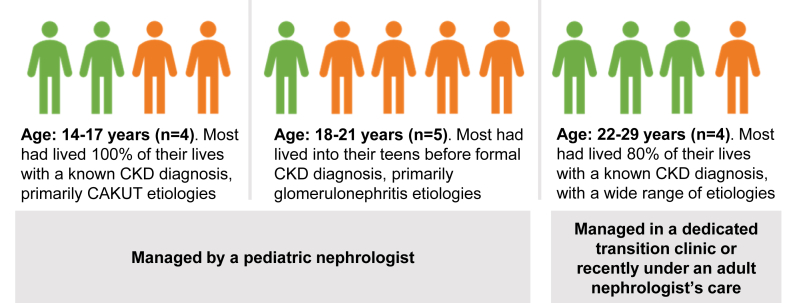
Characteristics of included patients. Orange figures represent females; green figures represent males. Abbreviations: CKD, chronic kidney disease; CAKUT, congenital anomalies of the kidney and urinary tract.

Twenty-three participants (13 patients and 10 parents) completed an interview. Four patients (31%) were <18 years of age (Fig 1). Most patients had CKD stage 4 (n = 8, 62%; 3 patients had CKD stage 5). There was a bimodal distribution in age at CKD diagnosis: 7 patients were diagnosed at birth or before 3 years of age, and 4 patients were diagnosed as teenagers (Table 1, Fig 1). The majority of patients either lived in a stable 2-parent household or had grown up with 2 parents while living at home (11 of 13). The 10 parents included 9 mothers (2 Spanish speaking) and 1 father; all but 2 participated in a dyadic interview with their child. Eight parents (80%) were in long-term relationships. At the time of interview, 9 patients (69%) did not have any known potential living donors, and 9 patients (69%) reported never having heard of PD from their health care providers. As of July 2025, about half of participants remain waitlisted and had not yet started dialysis (n = 6, 46%), and about half had received a transplant (n = 6, 46%; Table 2).

**Table 1:** Characteristics of Participants at Time of Interview^a^

Characteristic	Patients (n = 13)	Parents (n = 10)
Age (mean ± standard deviation, y)	20 ± 4 y	50 ± 10 y^b^
Under 18 years of age	4 (31%)	0 (0%)
Female	7 (54%)	9 (90%)
Chronic kidney disease characteristics		
Years living with chronic kidney disease (mean ± standard deviation, years)	14 ± 8 years	n/a
Glomerular filtration rate (mean ± standard deviation, mL/min/1.73m^2^)^c^	21 ± 8	n/a
Under pediatric nephrology care^d^	9 (69%)	n/a
Race/Ethnicity^e^		
Asian	1 (8%)	0
Black or African American	2 (15%)	0
White	7 (54%)	7 (70%)
Hispanic/Latino	3 (23%)	3 (30%)
Spanish as preferred language	0 (0%)	2 (20%)

a Data are presented as number (percentage) of individuals unless otherwise indicated.

b Eight parents reported their age using an optional demographic survey.

c As reported in most recent nephrology progress note at the time of recruitment or calculated using the Schwartz equation if not specifically reported in the note.

d Options included pediatric nephrologist, nephrologist with training in internal medicine and pediatrics, or adult nephrologist.

e Race/ethnicity was self-reported and either ascertained organically during interviews or through an optional demographics survey following the interview.

**Table 2:** Participants and Outcomes

Patient ID, Age Category, Sex as reported by patient	Parent ID, Relationship	CKD Stage^a^	CKD Etiology^b^	Outcome^c^
Patient 1, ≥18-year-old boy	n/a	5	GN	Received preemptive LUKT
Patient 2, ≥18-year-old boy	Parent 2, mother	4	CAKUT	Waitlisted, anticipating preemptive LUKT
Patient 3, ≥18-year-old boy	n/a	4	GN	Waitlisted, anticipating preemptive LRKT from father
Patient 4, ≥18-year-old girl	Parent 4, mother	5	Unknown	Received DDKT after a period of hemodialysis
Patient 5, ≥18-year-old girl	n/a	3B	CHC	Waitlisted, no known living donors
Patient 6, ≥18-year-old girl	Parent 6, mother	4	GN	Received preemptive DDKT
Patient 7, ≥18-year-old girl	Parent 7, mother	4	CHC	Received preemptive DDKT
Patient 8, ≥18-year-old boy	Parent 8, mother	4	CAKUT	Waitlisted, anticipating preemptive LUKT
Patient 9, ≥18-year-old girl	Parent 9, mother	4	CHC	Waitlisted, anticipating preemptive LRKT from mother
Patient 10, <18-year-old boy	Parent 10, mother	4	Other	Received preemptive DDKT
Patient 11, <18-year-old boy	Parent 11, mother	3B	CAKUT	Waitlisted, no known living donors
Patient 12, <18-year-old girl	Parent 12, mother	4	CAKUT	Received preemptive LRKT from sister
Patient 13, <18-year-old girl	Parent 13, father	5	CAKUT	Received DDKT after a period of hemodialysis

Abbreviations: CAKUT, congenital anomalies of the kidney and urinary tract; CHC, cystic/hereditary/congenital disorders as defined by the US Renal Data System; CKD, chronic kidney disease; DDKT, deceased donor kidney transplant; GN, glomerulonephritis; LRKT, living related kidney transplant; LUKT, living unrelated kidney transplant.

a At time of interview.

b Etiology defined using US Renal Data System categories to protect patient confidentiality, options include the following: CAKUT, glomerulonephritis, CHC, and other/unknown.

c As of December 2025, as derived from medical record documentation.

**Figure 2:**
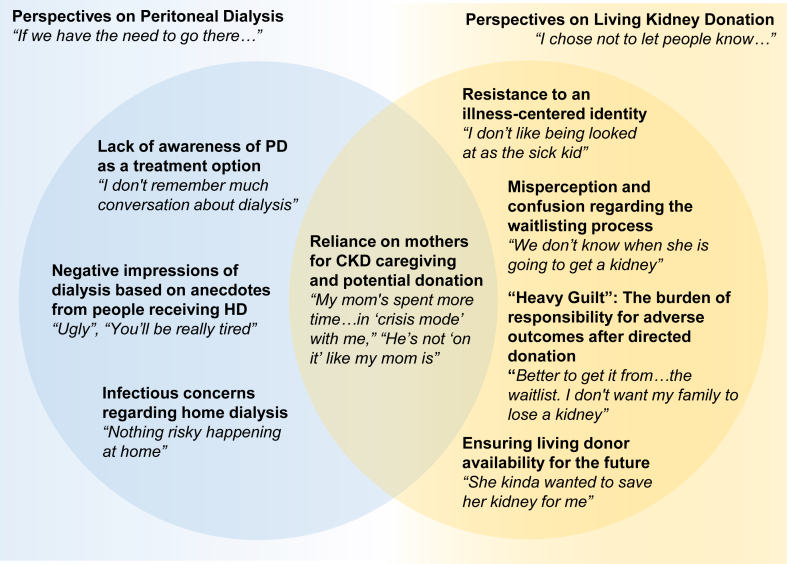
Depiction of identified themes.

Our study showed unexpected responses to our research question about how adolescents and young adults with CKD, and their parents, understood and appreciate KRT modalities. The main findings included low levels of awareness of PD and nuanced, complex perspectives on living donor kidney transplant. To contextualize these key findings, we present 8 themes, many of which serve as potential barriers to either PD or living donation. Perspectives on PD included the following: (1) lack of awareness of PD as a treatment option; (2) negative impressions of dialysis based on anecdotes from people receiving HD; and (3) infectious concerns regarding home dialysis. Perspectives on living donor kidney transplant included the following: (1) resistance to an illness-centered identity; (2) misperception and confusion regarding the waitlisting process; (3) “heavy guilt”: the burden of responsibility for adverse outcomes after directed donation; and (4) ensuring living donor availability for the future. These themes and an overlapping theme of reliance on mothers for CKD caregiving and potential donation are depicted in Figure 2 with exemplar quotations in Table 3.

**Table 3:** Perspectives on Peritoneal Dialysis and Living Donor Kidney Transplant

Theme: Lack of PD Awareness Coupled with Anecdotal Negative Impressions of HD	
Patient example quotation	Parent example quotation
Patient 10: “I don’t know much about [dialysis], so I can't really have a lot of input on that. Sorry about that…I definitely heard of the blood cleaning process.”	Parent 10: “I only know about the dialysis my father-in-law was on, and that was one with a catheter in his neck. But I don't know about different types of dialysis…The few people that I've known that were on dialysis. I know that it gives them life, but it wasn't the kind of life that I would want, especially for a young person.”
Patient 7: “I think I get a transplant or something [when my kidneys fail]. I think there was something called dialysis? I don't really remember.” Patient 7: “They [doctors] say that you'd have appointments and something. You'll always go, they'll hook you up to some machine or whatever. I think to your arm or something? That's about it, that I know [about dialysis].” Patient 7: “I did not hear about [PD]. That [relative on PD], I don’t really know him.”	Parent 7: “I know there's different hours, I guess, some that are longer than others?…I don't remember talking to her doctor about the different types of dialysis, to be honest with you…we don't know how many hours. We don't know where. We don't have all the details for that or have even heard the options.” Parent 7: “[Relative] had the overnight machine with him everywhere. So I've heard about that option [PD], but not from the doctors.”
Patient 9: “I only know about the one where you're hooked up to the machine, and then they have the port, and then they filter your blood. We haven't really focused a lot on dialysis, so I'm not sure…maybe [the doctors] feel like we have a lot more time to find donors or matches.”	Parent 9: “I don't remember much conversation about dialysis.”
Patient 4: “We haven't really talked about dialysis, just the doctors said that I have those two options [when my kidneys fail]…I can do the kidney transplant, or I can do the—what was that thing I forgot the name of? Yeah, dialysis.” Patient 4: “I heard people who do dialysis, they’re not able to drink a lot of water, as far as I know…if I have a kidney [transplant], I’m able to eat and everything, and be healthy.”	Parent 4: “I hope she never has to do dialysis. That is something I would not like them to have to do…Well to me it's ugly, that they would be changing blood every time.”
Patient 6: “I just know that dialysis is an option while waiting for a kidney… I heard [mom] mention [PD] when they were gonna start my grandma’s dialysis, they had mentioned that [PD] was one of the options…And my dad’s best friend used to be on dialysis, and I’ve kind of seen the sites…at least the people I’ve seen, you’ll be really tired, and they don’t work or anything.”	Parent 6: “I know of a dialysis that one has to go to the place where they do the dialysis…Every third day is like a washing machine for the blood, the machine gets connected, it runs, and it’s cleaning the blood…My mom has been undergoing [hemo]dialysis for two years.”
Theme: Infectious Concerns Regarding Home Dialysis	
Patient example quotation	Parent example quotation
Patient 13: “They're professional. And mom and dad are new to it.”	Parent 13: “At least [with PD], you know you can be with your friends, family. [With HD, the] benefits will be much better just by doing it with doctors around and stuff. Doing it at home would be more time with friends and family for her. But yeah, [we chose HD] just to feel like it's nothing risky happening at home when you're doing it.”
Patient 6: “…they wouldn’t know who would be in charge of cleaning it and making sure everything is sanitary.”	Parent 6: “I have heard that [PD] is…well, very delicate. You have to do a lot of cleaning and everything has to be really well disinfected so that they do not contract another disease. Because dialysis lowers one’s defenses too, I think that’s the case…if dialysis is necessary, it is better to have it done at the center.”
Theme: Resistance to an Illness-Centered Identity	
Patient example quotation	Parent example quotation
Patient 7: “My immediate family, the ones I live with [know about my CKD]. But everyone else that could apply and help out if they want to? They don't know…I chose not to let people know. I don’t like being looked at as the ‘sick kid.’ It doesn't seem like it's really their business…we're not that close.”	Parent 7: “[After she was diagnosed] in the beginning, she just really was in a really not-good place mentally…She just kind of felt like ‘that's it.’ Like her life's pretty much done with, right? Like she can't really do nothing. But that's not true at all. And we tell her that all the time.”
Patient 12: “[Once people know I need a transplant] people would be asking me constantly if I'm okay. Like ‘yes, I'm fine!’”	Parent 12: “I've asked [her] to not share with friends until we know more about what the [donation] process is…As long as she can just be a normal teen, the longer that we can maintain that, the better…We're trying to keep her medical stuff private for now…Asking for a kidney donation within the community? It will go ‘boom.’ I want [her] to have as much time without that label as possible.”
Theme: Misperception and Confusion Regarding the Waitlisting Process	
Patient example quotation	Parent example quotation
Patient 7: “I don't know about that part [getting a kidney from someone I know]…I'm pretty sure they put me on the list…of the kidneys.”	Parent 7: “She's been on the list from the beginning. I guess whenever a kidney comes?…We don't know, right? We don't know when she's going to get a kidney.”
Theme: “Heavy Guilt”: The Burden of Responsibility for Adverse Outcomes after Directed Donation	
Patient example quotation	Parent example quotation
Patient 9: “At one point I was like, ‘I don't even want to do it. I'd rather just not have a transplant because I don't even want to deal with the fact of losing [it].’ Someone gave me their organ, a part of their body, and I lose that?…That's heavy guilt.”	Parent 9: “I can appreciate her struggle with having a hard time receiving that gift from someone, and not feeling worthy of it…I don't know that I want someone to jeopardize their life to save mine.”
Patient 13: “Better to get it from somebody on the waitlist. I don't want my family to lose a kidney.”	Parent 13: “If someone is going to donate—for example, me—I want to make sure first that I'm healthy enough to do so… to have no side effects in the future. If something happened, [my daughter] would be like, ‘oh that was my fault.’”
Theme: Ensuring Living Donor Availability for the Future	
Patient example quotation	Parent example quotation
Patient 6: “My sister had said, since kidneys only last so long…if I could get one from the transplant list, it would be better since I'm younger and we've heard that younger people get higher priority. So if I couldn't get one from [the waitlist], she would donate it. She kinda wanted to like ‘save’ her kidney [for me, for later]. And if within a couple of years I need another transplant, since I'd be older I would be less priority.”	Parent 6: “Her sister would also like to give to her but she would like, as I said, to have the option first that someone else could donate. And if there isn't, she would be the second option.”
Theme: Reliance on Mothers for CKD Caregiving and Potential Donation	
Patient example quotation	Parent example quotation
Patient 9: “Honestly if my dad would have dealt with all these medical things, I think I would have been dead at this point.”	Parent 9: “…her dad's not knowledgeable.”
Patient 8: “My dad, sometimes he'll be involved. He's just not as good with it, I think. And he cares and he's really there for me, but my mom's just spent more time around doctors. She's spent more time in ‘crisis mode’ with me…I think for him it's a little bit more foreign, which stresses him out because he wants to help me, but sometimes he feels like he's not as well equipped, which he isn't.”	Parent 8: “[Father] had not been involved in hardly any of [patient]'s real day-to-day care. So he, even more so, was deer in the headlights [at the transplant evaluation]. At one point, the social worker asked him who he was! And he's like, ‘I'm the dad.’”

### Perspectives on PD

#### Lack of Awareness of PD as a Treatment Option

The majority of patients (n = 9) and parents (n = 7) did not recall ever hearing about PD from their nephrology providers. Two of the dyads had heard of PD from relatives who required KRT, but both dyads were unaware that PD could be an option for themselves or their child. Despite developing interview probes to inquire about non-HD forms of dialysis, many participants continued to voice “no, I have not” (Patient 2), “haven’t heard anything” (Patient 8), and “I don’t remember talking to her doctor about the different types of dialysis” (Parent 7). Only 3 of 13 patients had selected PD as their preferred dialysis modality “if we have the need to go there” (Parent 12), and all 3 remained hopeful for a preemptive transplant. In all cases where patients did have knowledge of PD, patients voiced PD was preferable to HD to prioritize consistent work or school attendance.

#### Negative Impressions of Dialysis Based on Anecdotes from People Receiving HD

Those who expressed familiarity with any form of dialysis usually derived their impressions from older relatives or family friends receiving in-center HD. Patients’ and parents’ both expressed negative sentiments toward HD (eg, “ugly,” Parent 4; “boring,” Patient 11). Patients and parents recalled anecdotes like “the dialysis my father-in-law was on…with a catheter in his neck . . . it wasn't the kind of life that I would want, especially for a young person” (Parent 10) and “my dad’s best friend used to be on [hemo]dialysis . . . you’ll be really tired” (Patient 6).

Furthermore, both patients and parents also expressed positive sentiments associated with being able to “avoid” (4 patients, 3 parents) or “skip” dialysis (Patient 2, Parent 12), such as “excited” (Parent 12) and “awesome” (Patient 5). These positive sentiments seemed to have been reinforced by their providers even when no living donor had been identified, eg, “Dr. [X] is pretty reassuring that [the need for dialysis] won’t happen” (Parent 11), and “we haven’t really focused a lot on dialysis…maybe [the doctors] feel like we have a lot more time to find donors” (Patient 9).

#### Infectious Concerns Regarding Home Dialysis

Two parents explicitly expressed a preference for HD and opposed PD because of concerns about infectious risks. Although a father acknowledged that PD could facilitate more time at home, HD would still be “much better” for his daughter to ensure there is “nothing risky happening at home” (Parent 13). One mother voiced that “if dialysis is necessary, it is better to have it done at the center… because of the things about the cleaning, or preventing infections” (Parent 6). This impression was derived from her own mother initiating HD at an elderly age and not based on information provided by the patient’s clinical providers.

### Perspectives on Living Donor Kidney Transplant

In addition to the barriers described below, a few enabling factors were identified during interviews. These included relief on identifying a potential living donor and participants’ trust in their nephrologists’ reassurance regarding the high likelihood of preemptive transplantation. However, these facilitators were less prominent compared with the barriers described.

#### Resistance to an Illness-Centered Identity

This theme highlights patients’ and parents’ reluctance to disclose CKD status broadly to their social networks because of concerns about being labeled as “sick.” Although not a direct barrier, this tendency to avoid disclosure limited opportunities to recruit living donors. Participants varied in their opinions as to whether and when to disclose the need for a kidney donor. Patients did not want to assume a “sick kid” identity and many chose to perceive themselves as “normal” (Table 3). Among parents, maintaining their child’s sense of normalcy was often rooted in the desire to have their child be “without that [sick kid] label” even if it was at the cost of living donor recruitment because “asking for a kidney donation within the community? It will go ‘boom.’” (Parent 12). Another parent described keeping their child’s CKD a “secret” (Parent 4) because of fears of negative social consequences, citing past experiences of being the subject of gossip.

#### Misperception and Confusion Regarding the Waitlisting Process

Nationally, pediatric patients (<18 years) can be waitlisted for kidney transplant once progressive CKD and impending kidney failure are confirmed, regardless of glomerular filtration rate. Adult patients can be waitlisted once glomerular filtration rate is less than 20 mL/min/1.73 m^2^ or dialysis is required. Although both patients and parents had an appreciation for the importance of being waitlisted before age 18 years to receive pediatric waitlist priority for deceased donation, both groups expressed misunderstandings regarding patients’ current waitlist status. Patients often deferred to their parents to provide details. Misunderstandings of patients’ eligibility to receive organ offers when patients were inactive on the waitlist often led to a false sense of security that a deceased donor kidney would become available soon and facilitated the belief that the search for living donors was less pressing. One mother confidently stated that her daughter would get a transplant “whenever a kidney comes” (Parent 7) even though the patient was not active on the waitlist and could not receive organ offers. Another mother described that “they already have her there, registered, but they don’t want to activate her” but acknowledged the opacity in the waitlist process (“I don’t know how that [organ offer process] works,” Parent 4). In some cases, a deceased donor kidney was felt to be a more time-efficient solution, eg, to receive a transplant “as soon as possible” (Parent 13) to reduce the patient’s exposure to dialysis.

#### “Heavy Guilt”: The Burden of Responsibility for Adverse Outcomes after Directed Donation

Although many participants were aware that living donor kidney transplants were associated with better graft survival compared with deceased donor kidneys,^1^ both patients and parents expressed varying degrees of anticipatory guilt and liability in receiving a kidney from a directed donor (when the living donor is known to the patient), which often translated into a stance that deceased donor transplantation was preferable. Parental feelings of not wanting others “sacrificing themselves” for the patient (Parent 9) were common. Patients reported feeling “uncomfortable” when it came to sharing the need for living donors with others because “I could slip up one time and it rejects…that’s heavy guilt” (Patient 9). As a result, some families delayed or avoided seeking living donors altogether and opted to wait for a deceased donor kidney.

#### Ensuring Living Donor Availability for the Future

Patients and parents were aware of the high probability that they (or their child) would need more than one kidney transplant in their lifetime and sometimes allowed their focus on barriers to a second transplant to interfere with the pursuit of a living donor for the first transplant. For example, a patient reported her adult sister “kinda wanted to ‘save’ her kidney [for me, in case] within a couple of years I need another transplant” (Patient 6). Many participants recalled a variety of other living donation options (eg, exchange program, voucher program), but could not provide additional detail on these programs.

### A Common Theme: Reliance on Mothers for CKD Caregiving and Potential Donation

Finally, we include a subtle finding that emerged organically during the interviews related to how family dynamics may shape KRT decisions in this population: 9 of 10 parents who participated were mothers. Patients generally described fathers and stepfathers as being less reliable and not knowing details of the patient’s care as compared with their mother, whether passively (“[My mom] has spent more time in ‘crisis mode’ with me,” Patient 8) or actively (“he tries his best,” Patient 5). Although half of participating mothers had initiated the donor work-up process, some already knew they were ineligible. Participants reported fathers generally did not attend appointments and did not participate in the family’s discussions regarding living donation. Instead, fathers assumed other important roles within families, for example as a “breadwinner” (Parent 7).

However, there were 2 examples in which fathers were more actively engaged in helping their child negotiate decisions around KRT (Patient 3; Parent 13). In both cases, we observed a notable positive impact on living donation. One father had already been medically cleared as the patient’s living donor, and one was interested in beginning the donor work-up.

## Discussion

This qualitative study identified multiple potential barriers to optimal forms of KRT among adolescents and young adults with nondialysis-dependent CKD and their parents. The predominance of barriers over facilitators in this study may reflect the concerns or fears of many participants’ with respect to KRT decision making. In particular, although many adolescents and young adults had lived with CKD for over a decade, these patients and their parents had limited awareness of PD as a form of KRT, and many were not motivated to recruit living donors.

Despite the expectation that most patients with longstanding CKD would be familiar with PD and HD as alternatives to transplant, we found a surprising and prominent awareness gap. Participants expressed an overall lack of awareness of PD as a KRT modality. This often stemmed from either (1) not recalling discussions of dialysis with their providers or (2) optimism on both the family and provider’s part that dialysis would be avoidable with a preemptive transplant. Although in truth, many participants ultimately did receive a first preemptive transplant and did not require dialysis, their lack of awareness of dialysis options contrasts sharply with participants’ high level of awareness around the need for multiple lifetime transplants. Participants and their families did not connect this fact with the high likelihood that they would also be likely to need dialysis in the future and should be aware of their treatment options. This aligned with observations from larger cohort studies of children with CKD, where 44%-51% of children (with similar CKD severity as included in our study) and their families reported a lack of recall of any discussions regarding KRT with their provider in the prior year. Two-thirds of those who recalled any KRT discussion remember only transplant—not dialysis—being discussed.^19^ In contrast, among older adults receiving KRT, 61% recalled PD being discussed as an option before initiation of long-term dialysis.^20^ Given that many teens with longstanding CKD (eg, because of congenital causes of kidney disease) may suffer from neurocognitive dysfunction,^21^ these patients may require iterative discussions regarding KRT options (HD, PD, and kidney donation). Furthermore, although lack of an engaged caregiver^22^ and lack of physical space in the home^23^ have been identified as barriers to PD among older adults, these themes were not identified in this study. Instead, patients and caregivers focused on their fears of infectious complications of PD as a source of their reluctance to choose this modality.

In some cases, knowledge of the need for a second transplant in the future detracted from the motivation to identify a living donor immediately, suggesting that long-term strategic planning by patients and families may actually be a barrier to living donor kidney transplant. Whereas most older adults may only receive one kidney transplant in their lifetime,^1^ the high likelihood of needing multiple lifetime transplants is unique to this younger population. Recent observational studies suggest that a living donor-first strategy is preferred for young patients with kidney failure.^24^ Providers should work to refute presumptions that “saving” a potential living donor for the future is of unequivocal benefit,^25^ as parents and older siblings may become medically ineligible for donation over time. Emphasizing the long-term benefits associated with a first transplant from a living donor could help optimize patient outcomes.

Some similarities were identified in terms of barriers to living donor kidney transplant in this age group, as compared with those previously identified among older adults with CKD, including misunderstandings related to the complicated organ allocation process,^26^ feelings of guilt in asking others to donate, and worry that the donor would experience negative outcomes or future side effects.^27^^,^^28^ Among older adults, more accurate knowledge of outcomes among living donors has been associated with greater likelihood of having a conversation about living donation with members of patients’ social networks.^27^^,^^29^ Thus, enhanced education about the safety of living donation may help allay fears of negative donor outcomes. However, because most living donor candidacy evaluation and work-up is performed by adult transplant centers, whether pediatric providers are providing the education and knowledge needed surrounding donor evaluation and outcomes is unclear.

### Strengths

A strength of this study was the number of parents participating alongside their child to provide dyadic perspectives; similar studies have typically included <10 caregivers (range, 0-23).^26^^,^^30^, ^31^, ^32^, ^33^, ^34^, ^35^, ^36^ In contrast to existing qualitative literature among young adults,^5^, ^6^, ^7^ participants had no prior personal history of either dialysis or kidney transplant. Thus, this work provides a unique perspective during a narrow window of time when a patient or family’s KRT decisions are still being shaped and minimizes recall bias that may occur when interviews are conducted with patients who have already initiated a form of KRT.

#### Limitations

This study included patients referred to one kidney transplant center in the San Francisco Bay Area. Because the kidney transplant process varies across the United States, findings may be context specific, but the identified themes are transferable to others with childhood-onset CKD. There was limited participation in our interviews by fathers, despite the majority of patients having grown up with, or still living in, a 2-parent household. This reflects a well-known trend of mothers serving as both the primary caregiver, and as research participants, for children with chronic health conditions^37^ including CKD.^38^

## Conclusions

This qualitative study found that adolescents and young adults, many of whom had lived with CKD for over a decade. Their parents had limited awareness of PD, and many were not motivated to recruit living donors. Some of the identified barriers to PD and living donation in this population are surmountable through increased education and reframing of discussions with their nephrology providers (eg, raising PD awareness, addressing potential benefits of living donor over deceased donor kidney transplant for a first transplant). High-quality patient-oriented materials to understand the pros and cons of different dialysis modalities^39^ and frameworks for making a living donation “ask” are readily available^40^ and could be tailored for use in this age group. Iterative education and counseling of families to ensure accurate information is retained may help address some of the potential barriers to PD and living donor kidney transplant in the adolescent and young adult population.

## Supplementary Materials

Supplementary File (PDF) Items S1, S2.
