# Supplementary material for: Perspectives on Peritoneal Dialysis and Kidney Transplant in Adolescents and Young Adults: A Qualitative Study
Source: Kidney Med. 2026 Mar 11;8(5):101316. doi: 10.1016/j.xkme.2026.101316 (PMC13097012; doi:10.1016/j.xkme.2026.101316)
Supplement: Supplementary File (PDF) — Items S1, S2. [file mmc1.pdf]

Supplementary Material

Item S1. Additional Consolidated Criteria for Reporting Qualitative Research (COREQ) Reporting Guidelines

Item S2. Interview Guide (Spanish & English)

Item S1. Additional Consolidated Criteria for Reporting Qualitative Research (COREQ) Reporting Guidelines

In concordance with COREQ reporting guidelines, the following information is provided:

Domain 1 (Research Team and Reflexivity):

All interviews were conducted by A.B. (practicing pediatric nephrologist, and certified bilingual Spanish/English). A.B. did not have a pre-existing clinical relationship with any participant, but in some cases had received information regarding eligibility and family dynamics from the referring provider, and briefly reviewed the patient's medical record to assess medical eligibility for participation. A.B. did not volunteer any personal biases or assumptions, but did share reasons for doing the research during the consent/assent discussion prior to commencing the interview. In the process of reaffirming confidentiality, A.B. made clear whether she knew or worked with the patient's primary nephrologist, but that their decision to participate (or not) was not and would not be discussed with that physician or affect their future medical care.

Domain 2 (Study Design):

Additional participant selection details. Minor patients chose whether to participate alone or with a parent, after parental assent. Two patient-parent dyads did not respond to interview requests, and one additional patient-parent dyad actively declined to participate (reason unknown). Of those whose parents participated but did not complete dyadic interviews, both patients were >18 years of age. Of the three patient-participants who opted not to invite a parent to participate, all were ≥23 years of age.

Setting. In-person interviews were conducted at UCSF Benioff Children's Hospital San Francisco and audio-recorded utilizing Zoom. Virtual interviews were audio- and video-recorded utilizing Zoom. Interviewing as a dyad was not compulsory; preferences were assessed prior to commencing the interview.

Data collection. The interview guide was not formally pilot tested, but was reviewed with E.K. (practicing pediatric and adult nephrologist and clinical researcher), and again with K.K. (medical anthropologist and qualitative researcher) after each of the first 3 patient interviews to ensure questions and responses were adequately addressing the proposed topic. No one was present during interviews besides the participants and A.B. Participants were not contacted for any follow-up interviews. A.B. wrote field notes after each interview with preliminary impressions. Transcripts were not returned to participants for comment or correction.

Domain 3 (Analysis and Findings):

Interviews were uploaded into MAXQDA and then transcribed verbatim by A.B. into English or Spanish within MAXQDA and manually deidentified. Translations of Spanish transcripts into English were performed by native speakers and/or certified bilingual providers (A.B. and G.A.R., a physician and nephrology researcher). Interpretive disagreements were discussed until a consensus was reached. Participants did not provide feedback on findings. Individual interviews ranged from 30-44 minutes, while dyad interviews were up to 75 minutes.

Analysis approach. This analysis was informed by the Sort and Sift qualitative analytic approach, which emphasizes an iterative shift from theory-informed expectations to data-driven interpretation (Maietta, 2021)[18], in combination with grounded theory principles. In the initial "Diving In" phase, analysts closely engaged with individual transcripts in addition to

establishing any explicit barriers and facilitators to home dialysis and living donation (when available/applicable), analysts identified a number of salient quotations, wrote analytic memos, created diagrams, and developed brief case profiles to allow key ideas, processes, and points of tension to emerge inductively. Observations were documented and iteratively refined as additional interviews were reviewed.

Rather than relying solely on static codebook refinement, analysis emphasized "topic monitoring," a dynamic, iterative process in which topics were tracked, refined, and integrated across interviews using analytic memos linking topics to supporting quotations. Topic integration took priority over enumeration of discrete codes. As analysis progressed, the team transitioned from within-interview analysis to cross-interview synthesis, examining relationships among topics and refining thematic interpretations.

In the subsequent "Stepping Back" phase, the analytic team examined patterns across transcripts through cross-interview comparison, linking and refining themes ("bridging and threading"), and documenting relationships between concepts. Analytic memos were revisited to identify connections, contrasts, and recurring processes that informed theme development and presentation. This iterative movement between close engagement with individual narratives and synthesis across interviews supported the development of coherent, data-grounded themes. Interviews continued until thematic saturation was reached, defined as two successive dyad interviews yielding no substantively new conceptual insights.

A.B. served as the lead analyst, reviewing and summarizing reports from all coders for all interviews, and presented preliminary interpretations and thematic linkages grounded in the data for discussion at each periodic team meeting. Co-authors independently reviewed subsequent memos and were encouraged to challenge, refine, or reject these interpretations through iterative team discussion until consensus was reached.

Item S2. Interview Guide and Excerpts (Spanish & English)

Introduction:
- Confirm pronouns, nicknames, relationship to and term used for caregiver
- Review assent/informed consent
- Confirm order of interview (dyad vs. individual)
- Reaffirm confidentiality, OK to skip questions or stop at any time

What do you know about what's going to happen when you/your child or loved one's kidneys fail or stop functioning well enough?
¿Qué sabe acerca de lo que sucederá cuando los riñones de su [hijo/ser querido] fallen o dejen de funcionar suficientemente bien?

How comfortable are you with your understanding of what happens when a person's kidneys stop working well, or they have "kidney failure"?
¿Qué tan cómodo se siente con su comprensión de lo que sucede cuando los riñones de una persona dejan de funcionar bien o tienen "insuficiencia renal"?
- Define "kidney failure" if necessary, clarify participants' understanding of whatever terms they have used, or terms they are familiar with. For example, "this is usually once your kidneys have ≤15% function, once the glomerular filtration rate or "GFR" is less than 15 or 20 mL/min/1.73 m^2, once your/their creatinine is very high, once your kidney can't clean your blood well, once kidneys don't make enough urine, to the point of needing a machine or another person's kidney (transplant) to take over for your own kidney(s)"
- *Esto usualmente es cuando sus riñones tienen función ≤15%, una vez que la tasa de filtración glomerular o TFG"(o "GFR" en ingles) es menos que 15 o 20 ml/min/1.73 m^2, cuando su creatinina es muy alta, que su riñón no sirve o no puede limpiar bien su sangre, que los riñones no producen suficiente orina, hasta el punto de necesitar una máquina o el riñón de otra persona (trasplante) para reemplazar su propio riñón(es)*

Can you share with me your thoughts about what happens at that stage?
¿Puedes compartir conmigo tus pensamientos sobre lo que sucede en esa etapa?

What options do people have at that point?
¿Qué opciones tiene la gente en ese momento?
- What can you tell me about what dialysis is or the different kinds of dialysis?
  ¿Qué puede decirme sobre qué es la diálisis o los diferentes tipos de diálisis?
- What can you tell me about what you know about the benefits of kidney transplant? What can you tell me about the different types of kidney transplant?
  ¿Qué puede decirme sobre lo que sabe sobre los beneficios del trasplante de riñón y los diferentes tipos de trasplante de riñón?

Probes regarding peritoneal dialysis: dialysis that is performed "at home," using a catheter "in the belly area," performing dialysis "every day" or "not at a center."

Do you know when that might be (kidney failure for you/loved one)?
¿Sabe cuándo podría ser eso (insuficiencia renal para usted/un ser querido)?

In your case, what kidney replacement therapy might you use? How was that decision made?
En su caso, ¿qué terapia de reemplazo renal podría utilizar? ¿Cómo se tomó esa decisión?

- (if they have decided) What helped you to make the decision?
  (si lo han decidido) ¿Qué te ayudó a tomar la decisión?
- (if they haven't decided) What would be helpful for you in order to make the decision?
  (si no lo han decidido) ¿Qué le ayudaría a usted a tomar la decisión?
- Who else was involved for those discussions? How did those conversations go?
  ¿Quién más estuvo presente en esas discusiones? ¿Cómo fueron esas conversaciones?
- Who helped you understand advantages and disadvantages regarding treatment options for your [child/loved one's] kidney failure? How did those conversations go?
  ¿Quién le ayudó a comprender las ventajas y desventajas con respecto a las opciones de tratamiento para la insuficiencia renal de su [hijo/a]? ¿Cómo fueron esas conversaciones?
- Were there situations when you didn't feel fully listened to, either by your child/caregiver, or by your medical team? Why do you think or feel that was the case?
  ¿Hubo situaciones en las que no se sintió completamente escuchado, ya sea por su hijo/cuidador o por su equipo médico? ¿Por qué cree o siente que fue así?

Based on responses to above, as appropriate, either in dyad interview or in private:

- Are you [caregiver] considering donating? Why or why not?
  ¿Está usted [cuidador] considerando donar? ¿Por qué o por qué no?
- What worries did you have about [modality they did not choose]?
  ¿Qué preocupaciones tenía sobre [modalidad que no eligieron]?
- What worries might you have about [chosen form of KRT]?
  ¿Qué preocupaciones podría tener sobre [forma elegida de KRT]?

Sub-questions may include, either in dyad interview or in private:

- Who are some other adults in your [family's] life that you're close with?
  ¿Quiénes son algunos otros adultos en su vida [familiar] con los que tiene una relación cercana?
- Have/had you thought about people in your life that might be able to donate? Why or why not?
  ¿Ha pensado en personas en su vida que podrían ser capaces de donar?
- Have you shared information about living donation with people in your life? What worries did you have about talking to them about this? How did those conversations go?
  ¿Ha compartido información sobre la donación en vida con personas en su vida? Qué preocupaciones tenía acerca de hablar con ellos sobre esto? ¿Cómo fueron esas conversaciones?

What else do you think might be important to add or mention about living with kidney disease? What others questions do you have?
¿Qué más cree que podría ser importante agregar o mencionar acerca de vivir con una enfermedad renal? ¿Qué otras preguntas tienes?

Can you tell me how [your loved one] having kidney disease has affected your life so far?
¿Puede decirme cómo la enfermedad renal de [su ser querido] ha afectado su vida hasta ahora?

Sub-questions to be addressed may include:

- What do you know about the current state of your [loved one's] kidney disease?
  ¿Qué sabe sobre el estado actual de la enfermedad renal [de su ser querido]?
- What's your relationship like with [caregiver], when it comes to your kidney disease?
  ¿Cómo es su relación con [cuidador], cuando se trata de su enfermedad renal?
- How do medical decisions, in general, get made in your household?
  ¿Cómo se toman las decisiones médicas, en general, en su hogar?
- What decisions were easy? Hard?
  ¿Qué decisiones fueron fáciles? ¿Duro/dificil?

What other thoughts do/did you have about how you [and/or your loved one] make decisions about [your] kidney care?
¿Qué otros pensamientos tiene/tuvo sobre cómo usted [y/o su ser querido] toman decisiones sobre el cuidado de [su] riñón? ¿Cómo fue esta experiencia para ti?

Conclusion:

- Provide resources to participants at end of interview, if interested (National Kidney Foundation website "Choosing Dialysis" and/or UCSF Living Kidney Donor Program, UCdonor.org)

Example of interview domains mapped to questions with excerpted responses

Domain	Interview Guide Excerpt	Direct Response Excerpt
Comprehension and expectations surrounding treatment options for kidney failure	What do you know about what's going to happen when you/your child or loved one's kidneys fail or stop functioning well enough?<br><br>What options do people have at that point?	"As far as what the doctor explained, we are waiting until her function drops to 15%, then she would be put on the waiting list." (Parent 6)<br><br>"I have two options, I can do the transplant or I can do…what was that thing I forgot the name of? Yeah, dialysis." (Patient 4)
Attitudes toward the dyad's preferred dialysis modality, if chosen	In your case, what kidney replacement therapy might you use? How was that decision made?<br><br>What can you tell me about what dialysis is, or the different kinds of dialysis?	"I really just want to be able to work…[PD] will just be easier to not fully up-end my entire schedule every day" (Patient 1)<br><br>"I don't know much about it, so I can't really have a lot of input on that, sorry." (Patient 10)
Attitudes toward transplant and living donation	What can you tell me about the different types of kidney transplant?	"Obviously there's, I don't know if they call them 'cadaver' ones, but from people that have passed away, that would always be kind of sad. And then people who want

		to donate." (Parent 11)
	Are you/your caregiver considering donating—why or why not?	"Yeah [my parents] are both too old, they're in their 60s." (Patient 5)
	Have you shared information about living donation with people in your life? How did those conversations go?	"I don't share it a lot because I think I'm just uncomfortable with this subject. I know my mom really does [share]." (Patient 9)<br>"Not really, that was kind of what my parents did." (Patient 2)

Developed by Alexandra Bicki, Kimberly Koester, Elaine Ku, March 2023.
